# A Large and Deep Root System Underlies High Nitrogen-Use Efficiency in Maize Production

**DOI:** 10.1371/journal.pone.0126293

**Published:** 2015-05-15

**Authors:** Peng Yu, Xuexian Li, Philip J. White, Chunjian Li

**Affiliations:** 1 Department of Plant Nutrition, College of Resources and Environmental Science, China Agricultural University, Beijing, 100193, China; 2 Ecological Sciences, The James Hutton Institute, Invergowrie, Dundee, DD2 5DA, United Kingdom; Agricultural Research Service, UNITED STATES

## Abstract

Excessive N fertilization results in low N-use efficiency (NUE) without any yield benefits and can have profound, long-term environmental consequences including soil acidification, N leaching and increased production of greenhouse gases. Improving NUE in crop production has been a longstanding, worldwide challenge. A crucial strategy to improve NUE is to enhance N uptake by roots. Taking maize as a model crop, we have compared root dry weight (RDW), root/shoot biomass ratio (R/S), and NUE of maize grown in the field in China and in western countries using data from 106 studies published since 1959. Detailed analysis revealed that the differences in the RDW and R/S of maize at silking in China and the western countries were not derived from variations in climate, geography, and stress factors. Instead, NUE was positively correlated with R/S and RDW; R/S and NUE of maize varieties grown in western countries were significantly greater than those grown in China. We then testified this conclusion by conducting field trials with representative maize hybrids in China (ZD958 and XY335) and the US (P32D79). We found that US P32D79 had a better root architecture for increased N uptake and removed more mineral N than Chinese cultivars from the 0-60 cm soil profile. Reported data and our field results demonstrate that a large and deep root, with an appropriate architecture and higher stress tolerance (higher plant density, drought and N deficiency), underlies high NUE in maize production. We recommend breeding for these traits to reduce the N-fertilizer use and thus N-leaching in maize production and paying more attention to increase tolerance to stresses in China.

## Introduction

Increased crop yields are predicted with the application of nitrogen (N) fertilizers, and it is estimated that about half of global food production is increased directly by application of N fertilizers [1–3]. However, over-use of N fertilizers can have serious environmental consequences, including N enrichment in natural ecosystems such as nitrate contamination in the groundwater and consequent changes in biodiversity and the emission of greenhouse gases, such N_2_O [4–9]. Increasing N-use efficiency (NUE) has been an important focus of recent agricultural and environmental researches [4, 10]. China is a major producer and consumer of N fertilizers. A 271% increase in N fertilizer application from 7.07 to 26.21 MT (million tons) has resulted in a mere 71% increase in annual crop grain production, (from 283 to 484 MT), over the past three decades [11], while NUE decreased from 55 to 20 kg kg^-1^ N [11, 12]. For maize, NUE in China decreased from 30.2 to 29.9 kg grain kg^-1^ N between 1980 and 2010. By contrast, maize NUE increased from 39.4 to 53.2 kg grain kg^-1^ N in the US during the same period (FAO 2012). Optimizing the management of N-fertilizer is extremely important for improving NUE. According to data from 66 experiments across thirteen major maize production provinces in China, integrated soil-crop management increased the NUE of maize to 57 kg kg^-1^ N with an average grain yield of 13 t ha^-1^ [13]. Using a similar approach, the average NUE of maize in Nebraska reached 73 kg kg^-1^ N with an average grain yield of 13.2 t ha^-1^ [14]. Apparently, a gap in NUE of maize production exists between China and western countries.

An essential strategy to improve NUE is to enhance N uptake by crops through breeding for appropriate root traits [15–17]. Efficient N uptake depends on root/shoot ratio (R/S), root size, and root distribution in the soil profile, which not only maximize interception and uptake of N fertilizers but also reduce N losses to deeper soil layers and groundwater, thereby increasing NUE [17, 18]. Water and nutrients uptake by crops depends on root branching in the top soil and root growth angle respectively [18]. Axial roots are able to exert greater forces on soil and might have greater ability to penetrate compact soil [19, 20] which determine growth directions and spatial distribution of a root system. Higher root length density (RLD) reflects greater ability of the root system [21] for increased water and nutrient uptake.

Although roots can play a significant role in increasing crop yields [22, 23], limited attention has been paid to root characteristics in crop breeding, possibly because roots grow belowground and are difficult to investigate [16, 23]. Over the past decades, improvement in maize’s ability to enhance root growth has been the primary driving force for higher yields of newer hybrids [24]. Plant breeding has contributed to 40–50% increased maize yield in the US [25] and 36% in China [26]. Plant breeders have made considerable gains on ‘aboveground’ traits, but little attention has been paid on root characteristics. Improvements in root dry weight (RDW) have been negatively reinforced to a certain extent by the high-yielding shoot traits [27]. Root sizes of new wheat cultivars are small compared with local varieties, which may limit water and nutrient uptake [28]. In China, the new varieties have larger RDW at silking [29]. However, the RLD of both old and new varieties in the 0–60 cm soil profile were similar, when grown under the same condition [30].

We investigated the relationships between R/S, root size, root architecture, and NUE in maize and compared the differences in maize varieties bred in China and western countries using data published in the past decades. Comprehensive analysis of the effects of climatic and geographical stress factors on the RDW and R/S, and NUE in maize production at silking in China and western countries was also implemented. To validate conclusions from the literature, a two-year field experiment with three maize varieties from China and US was conducted under the same growth conditions. The aim of this study was to answer the questions: are there differences in RDW and R/S of maize bred in China and western countries, and how these differences affect maize NUE?

## Materials and Methods

### Collection of data from previous literature

A literature search was performed using three electronic databases: Web of Science, Google Scholar, and CNKI (China National Knowledge Infrastructure). Data extracted from raw tables directly or transformed by calculation from the original figures on RDW and R/S of plants during the growing season were obtained from 106 studies (66 performed in China and 40 performed in western countries) published since 1959 that reported root characteristics of maize varieties grown in the field (listed in Table A in S1 Information). These studies were published in 53 different journals (35 in Chinese and 18 in English) and two conference proceedings. The field studies in China were performed in five major maize production areas, with different geographical (climatic) conditions and soil types (Fig. A in S1 Information): Region I is northeast China (19 experiments) with the humid climate of the middle latitude temperate zone and typical black or dark brown earth with high soil fertility. The northeast China Plain located between 39–53°N is one of the most important areas for spring maize production. The maize yield produced in this area contributes 35% of the total production in China [31]. Region II is north China Plain (18 experiments) with a humid and semi-humid areas of the warm temperate zone where brown earth is widely distributed. The north China Plain is one of the most intensive agriculture regions in China [12]. Region III is in Loess Plateau northwest China (13 experiments) with temperate continental climate and aeolian soils or gray-brown desert soils of lower soil fertility. Region IV is southwest China (6 experiments) with the subtropical monsoon climates and red and purplish soil. Specifically, we separated the data of Shandong province from the north China Plain as the Region V (east China, 10 experiments) due to larger amounts of chemical fertilizer application in this province compared with other regions.

**Fig 1:**
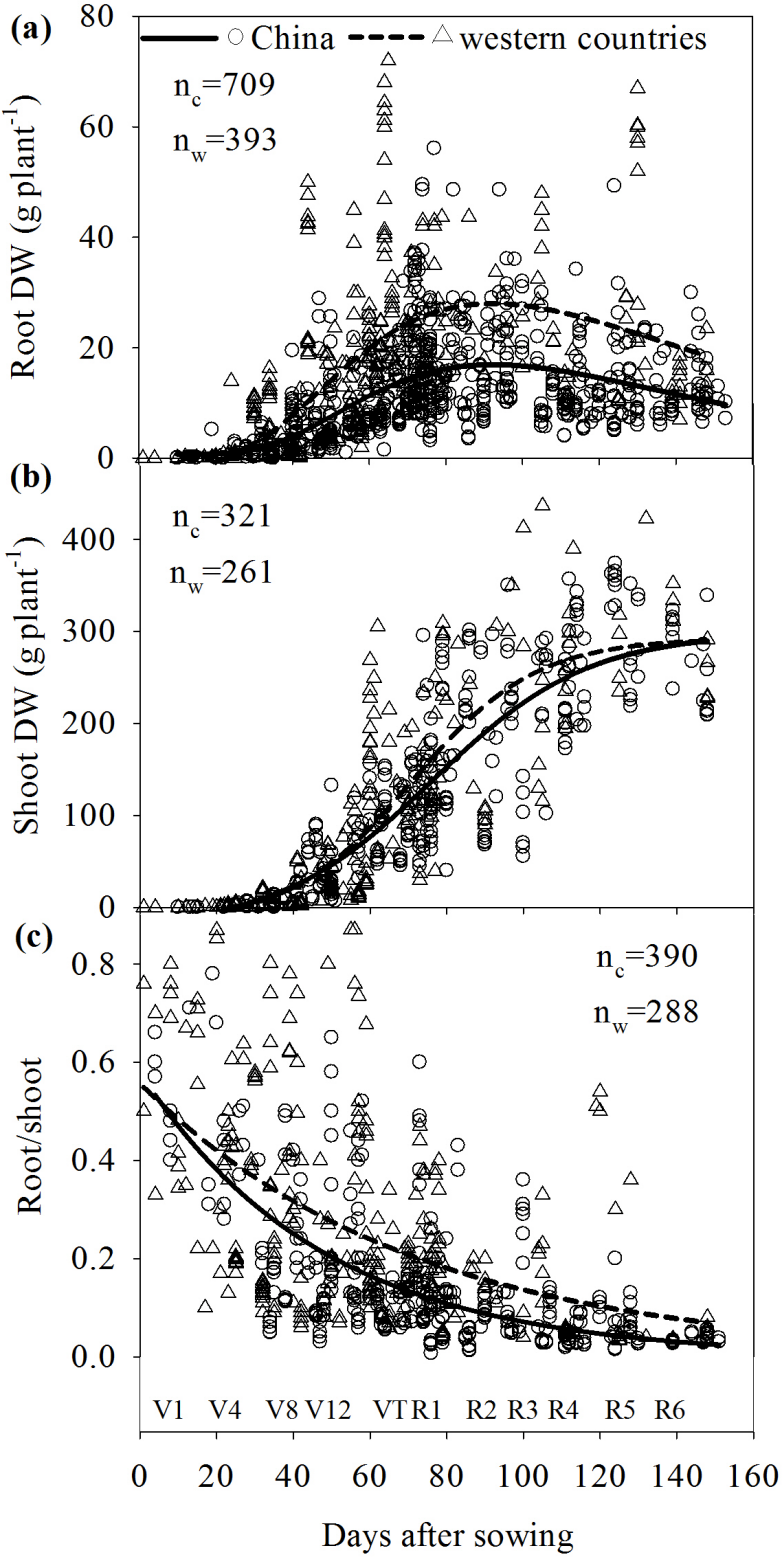
Comparison of the root and shoot dry weights and root/shoot ratio of maize varieties grown in China and western countries during maize growth. V1-V12: the first—twelfth leaf emerged with ligule visible; VT: tasselling; R1: silking; R2: grain ‘blister’ stage; R3: milk; R4: dough; R5: dent visible; R6: physiological maturity. Unpaired data in the scatter plots were collected from 106 publications studying maize root growth in field experiments over the last 50 years.

The field studies in western countries were performed in the US Corn Belt, that includes Iowa, Illinois and parts of Indiana and Nebraska (24 studies), Canada (three studies), Mexico (one study), and European countries (six from France, three from Germany and three from other countries). In total, 709 data were used to determine relationships between RDW and days after sowing (DAS) in China and 393 data in western countries (Fig 1A); 321 and 261 data from China and western countries, respectively, were used to determine relationships between shoot dry weight (SDW) and DAS (Fig 1B); 390 and 288 data from China and western countries, respectively, were used to determine relationships between R/S and DAS (Fig 1C).

**Fig 2:**
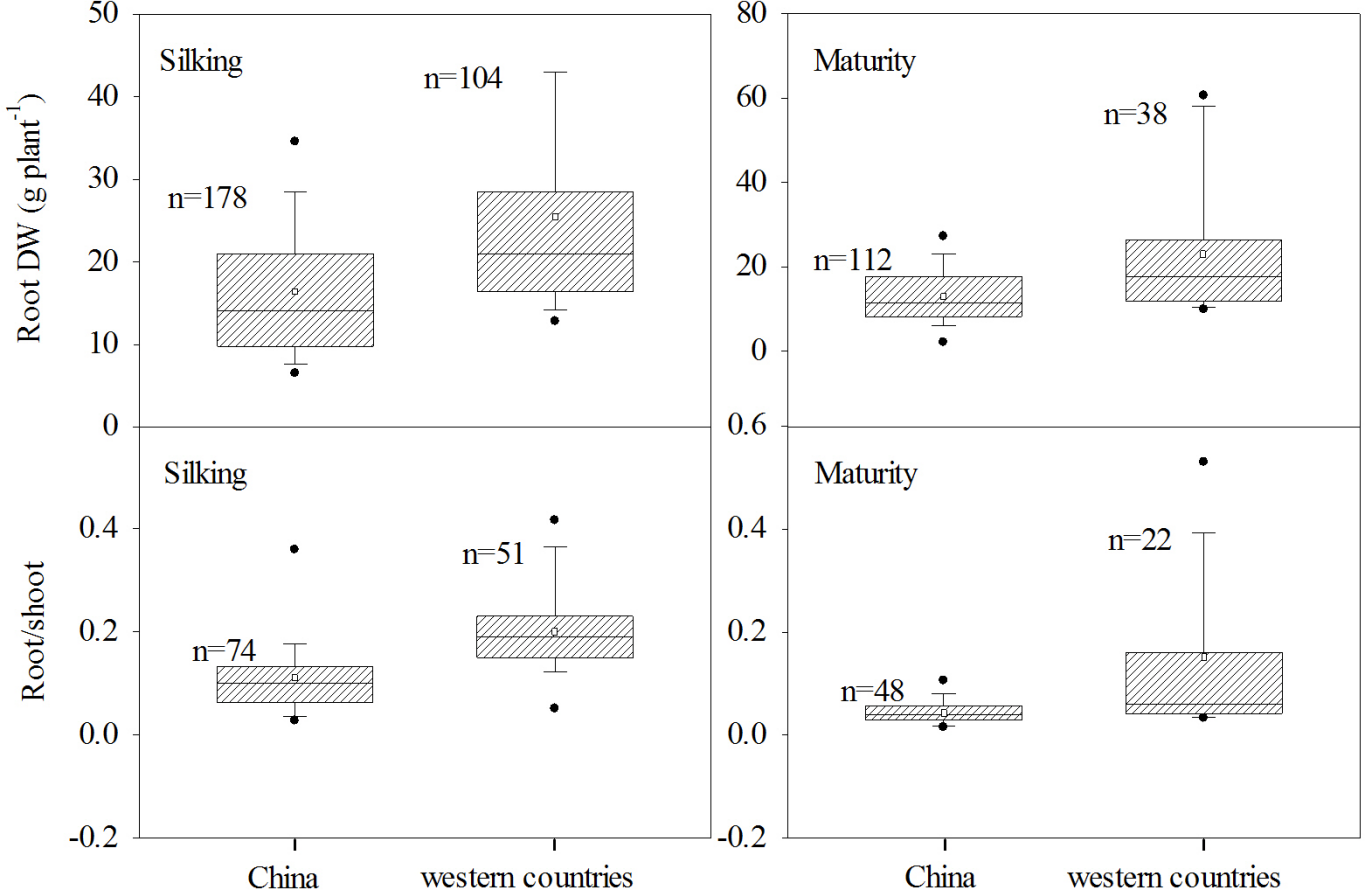
Root dry weight and root/shoot ratio at silking and maturity in unpaired data derived from field experiments using maize varieties in China and western countries. The solid line and square within the box represents the median and mean values of all data, the top and bottom edges of the box represent 75 and 25 percentiles and the top and bottom bars represent 95 and 5 percentiles of all data, respectively.

Data for RDW and R/S at silking and physiological maturity for maize grown in China and western countries were compared (Fig 2) based on data from 106 publications. Also data for RDW at silking for the selected four dominant Chinese maize varieties released in different years, ZD2 (1970s), YD13 (1980s), ZD958 and XY335 (two currently popular varieties) and one US pioneer were compared. Average changes in silking RDW of maize from China and US Corn Belt in response to high planting density, water stress and N deficiency were also compared based on the data from publications.

Only data generated from the field experiments were collected; those from glasshouses or growth chambers were excluded. Roots were generally harvested by destructive methods (see below). Data were pulled from experiments on different maize varieties, grown with contrasting planting densities, nutrient regimes, water supplies, tillage systems and other aspects of crop management on different soil types. Thirteen field studies without detailed experimental records or that did not conform to a logistic growth model were excluded from the study.

The R/S ratio was defined as the ratio of belowground dry biomass (root) to the aboveground dry biomass (stem, leaves, and husks plus grain). RDW and R/S values were obtained either directly or calculated from the published data. Sampling dates and growth stages (estimated from V1 to R6) were integrated into the same scale: DAS, calculated from the detailed information (planting, sampling and harvest time) in the corresponding papers, because different varieties have different duration of growth. The RDW was normalized as g plant^-1^ by dividing per unit root biomass by planting density [32]. All data for SDW were obtained from studies that contained RDW and R/S. Nitrogen deficiency, water stress, and planting density were considered as stress factors to compare the differences in RDW and R/S between China and the US Corn Belt.

### Field experiments to validate conclusions from literature analysis

The field experiments were conducted in two consecutive years (2011 and 2012) at the Shangzhuang Experimental Station (40^o^8′20″N, 116^o^10′47″E), China Agricultural University, Beijing. The soil type at the study site was a calcareous alluvial soil with a silt loam texture (FAO) typical of the region. The characteristics of the soil were analyzed prior to sowing. The chemical properties of the 0–30 cm soil layer were as follows: extracted mineral N (N_min_ = NH_4_^+^ + NO_3_^-^) 14.8 mg kg^-1^, pH (H_2_O) 8.0, soil bulk density 1.33 g cm^-3^, Olsen-P 17.5 mg kg^-1^, NH_4_OAc-extracted K 157.5 mg kg^-1^, and organic matter 21 g kg^-1^ in 2011; extracted mineral N (N_min_) 12.6 mg kg^-1^, pH (H_2_O) 8.0, soil bulk density 1.33 g cm^-3^, Olsen-P 18.5 mg kg^-1^, NH_4_OAc-extracted K 164.3 mg kg^-1^, and organic matter 20 g kg^-1^ in 2012. Three maize varieties (ZD 958 and XY 335 from China and Pioneer 32D79 from the US) were sown on 29 April in 2011 and 25 April in 2012. The amount of rainfall during the maize growing season and additional data from 2007 to 2010 were shown in Table B in S1 Information. In addition, 12 and 15 mm of irrigation were applied on 19 and 30 June 2011 and 44 mm on 15 June 2012.

Flood irrigation before plowing was used to maintain the available soil water content in the soil ranging from 100 to 200 mbar. One week later, the field was plowed just before sowing. Maize was over-seeded with hand planters and thinned at the V5 stage (the fifth leaf emerged with the visible ligule) to a stand of 70,000 plants ha^-1^. The distance between plants was 20 cm for intra-rows and 70 cm for inter-rows in 2011 and 28 cm for intra-rows and 50 cm for inter-rows in 2012. A randomized complete block design with four replicates for each treatment (total 12 plots) in each year was used. The plot size was 31.5 m^2^ (5 m × 6.3 m) in both years. Before sowing in 2011 and 2012, 60 kg N ha^-1^ as urea, 59 kg P ha^-1^ as triple superphosphate (Ca(H_2_PO_4_)_2_ H_2_O), 66 kg K ha^-1^ as potassium sulfate (K_2_SO_4_) and 7 kg Zn ha^-1^ as ZnSO_4_ 7H_2_O were broadcast and incorporated into the top 0–15 cm soil layer with rotary tillage. In 2011, 100 kg N ha^-1^ at V8 and 40 kg N ha^-1^ plus 40 kg K ha^-1^ at VT (tasselling) were applied by hand as topdressing. In 2012, 60 kg N ha^-1^ at V8 and 80 kg N ha^-1^ plus 40 kg K ha^-1^ at V12 were applied by hand as topdressing. Weed growth on plots was controlled by pre-emergence herbicides and cultivation.

### Plant harvest and measurements of dry weight and nitrogen content

Maize was harvested at silking (76 DAS in 2011 and 82 DAS in 2012) and at physiological maturity (148 DAS in 2011 and 153 DAS in 2012 when over 50% of plants showed a visible black layer at the base of the kernel). At each harvest, five consecutive plants were cut at the stem base in each plot. The shoot was divided into five parts: middle leaves (3 leaves, one ear leaf and one leaf above and one leaf below ear leaf), upper leaves (all the leaves above middle leaves), lower leaves (all the leaves below the middle leaves), stalks and ears. To determine the grain yield, ears in an area of 15.7 m^2^ (4.9 × 3.2 m) in each plot were hand-harvested at physiological maturity in both years. Kernels from six randomly selected ears were harvested individually by hand, and weighed to calculate grain yield at 15.5% moisture.

After shoot excision, roots from three plants were excavated from each plot. Roots from each plant were excavated with a soil volume of 20 cm (10 cm on each side of the plant base in the intra-row direction) × 70 cm (35 cm on each side of the plant base in the inter-row direction) × 40 cm in depth in 2011, and with a soil volume of 28 cm (14 cm on each side of the plant base in the intra-row direction) × 50 cm (25 cm on each side of the plant base in the inter-row direction) × 40 cm in depth in 2012. The sampling depth for RDW was determined according to the reports which suggested that 80–90% of the total RDW is distributed in the top 0 to 20 cm soil layer [33, 34]. The area of 20 cm × 70 cm in 2011 and 28 cm × 50 cm in 2012 was the soil surface occupied by each plant at the planting density of 70,000 plants ha^-1^. All of the visible roots in each excavated soil volume were picked out in the field by hand and washed with water to remove soil particles. All shoot and root samples were dried at 70°C for at least three days until constant weight was obtained. Dried samples were ground to 1 mm and 0.2 g of the ground plant material was used to determine the N concentration using a modified Kjeldahl digestion method [35]. Nitrogen content in aboveground plant sample was determined by multiplying N concentration by their dry weights and total N uptake was calculated as the sum of N content in all aboveground plant parts. NUE was determined by dividing the grain yield by N fertilizer input.

### Root and soil sampling using the monolith method

In order to study the spatial distribution of maize roots of the three varieties in the soil, the monolith method was used to obtain root and soil samples after shoot excision at silking when the maize had largest root system [36, 37]. Three replicates for each variety in each year were used for the study.

Soil cubes with 10 × 10 × 10 cm sides (1000 cm^3^) were dug one by one in a soil volume of 70 cm × 30 cm × 60 cm in depth in 2011, and 50 cm × 30 cm × 60 cm in depth in 2012. The total number of monoliths for each plant was 126 in 2011 and 90 in 2012, respectively. All visible roots in each soil monolith were picked out in the field by hand and placed in individual plastic bags, then marked with spatial coordinates. All roots picked out from each soil monolith were brought back to the lab, soaked in water, stirred, and poured into a 0.25 mm mesh sieve. The sieve was suspended in a large water bath and shaken continuously until all roots were washed free of the soil. Remaining soil materials on the sieve were removed by hand. The separated root fractions were scanned immediately, or kept at -20°C for subsequent scanning, with a scanner (Epson 1680, Indonesia). The scanned images were analyzed using WinRHIZO V5.0 (Regent Instruments Inc., Quebec City, Canada) [38]. RLD was the ratio of total root length to soil volume in each soil monolith. The RLD and soil N_min_ were presented as contour diagrams generated using Surfer 7.0 (Golden Software Inc., USA, 2000).

After picking visible roots, the soil in each monolith was crushed by hand and sieved through a 3 mm mesh sieve in the field, grinded using mortar and pestle in the lab to determine mineral nitrogen concentration (N_min_). A portion of the mixed soil was taken and placed in a marked plastic bag, and extracted immediately after transfer to the laboratory by shaking for 1 h at 25°C with 0.01 mol L^-1^ CaCl_2_ (1: 20, soil: water). Soil N_min_ (NH_4_^+^-N + NO_3_^−^N) was analyzed by continuous flow analysis (TRACS 2000 system, Bran and Luebbe, Norderstedt, Germany). The RLD and soil N_min_ were presented as contour diagrams generated using Surfer 7.0 (Golden Software Inc., USA, 2000).

### Statistical analysis

#### Data-mining analysis

Temporal variation of collected data in RDW, SDW, and R/S over the maize growing season (0–160 DAS) followed a nonlinear trend. Therefore, a nonlinear regression model (Sigmaplot 12.0, Systat Software Inc., Chicago, USA) was adopted to analyze the data (Fig 1). Coefficients were generated and *P* values were calculated at the significant level 0.05 using the Sigmaplot 12.0.

The equation used to describe the relationship between RDW and DAS was:

$$RDW={\text{y}}_{0}+a\times e^{-0.5{\left(\frac{DAS-x_{0}}{b}\right)}^{2}}$$

The coefficients were significant with *P* values < 0.0001 for parameters a, b and x_0_. The coefficients y_0_, a, b, and x_0_ were -10.3, 27.5, 57.2, 80.5 and standard errors of coefficients were 7.4, 7.2, 11.5 and 1.7 for China. For western countries, coefficients y_0_, a, b, and x_0_ were -11.6, 41.6, 50.4, 80 and standard errors were 7.5, 6.8, 8.7, 2.4. Results for the overall best-fit solution were R^2^ = 0.32 for China and R^2^ = 0.40 for western countries respectively.

The equation used to describe the relationship between SDW and DAS was:

$$SDW=y_{0}+a\times \left[1+e^{-\left(\frac{DAS-x_{0}}{b}\right)}\right]$$

The coefficients were significant with *P* values < 0.0001 for parameters a, b and x_0_. The coefficients y_0_, a, b, x_0_, were -23.4, 322.5, 20.4, 76.3 and standard errors of coefficients were 18.4, 30.9, 3.4 and 2.7 for China. For western countries, coefficients y_0_, a, b, and x_0_ were -18.2, 310.9, 16.2, 71 and standard errors of coefficients were 11.5, 23.1, 2.5 and 2.3. Results for the overall best-fit solution were R^2^ = 0.75 for both China and western countries.

The R/S was fitted to an exponential decay curve:

$$R/S=a\times e^{-bDAS}$$

The coefficients were significant with *P* values < 0.0001 for parameters a, b, with respective values of 0.58, 0.02 and standard errors 0.03, 0.001 for China. For western countries, values for a and b were 0.56, 0.01 and standard errors 0.04, 0.002. Results for the overall best-fit solution were R^2^ = 0.45 for China and R^2^ = 0.24 for western countries. Parameter a gives the initial R/S at maize seedlings emergence and R/S decreases exponentially as DAS increases.

The RDW and R/S ratio of maize grown in China and western countries were compared at silking and maturity using unpaired two-tail Student’s *t*-test (Sigmaplot 12.0, Systat Software Inc., Chicago, USA) (Fig 2). The difference was significant at a *P* value < 0.05.

Detailed analyses were performed to compare the differences in silking RDW and R/S ratio of varieties, planting regions and responses to different stress factors of maize from China and US pioneers.

#### Statistical analysis of field experiments

Field data on RDW, R/S, N uptake, yield and NUE were analyzed using one-way PROC ANOVA using the SAS package (SAS-Institute-Inc., Cary, NC, USA, 2004). Genotype treatments were treated as fixed effects and replication as random effect. Means of different genotype treatments were compared based on least significant difference (LSD) at the significance level of 0.05. Year was treated as repeated measure of analysis for data analysis on parameters shown in Table 1.

**Table 1:** Root dry weight (RDW) and root/shoot ratio (R/S) at silking (76 DAS in 2011 and 82 DAS in 2012), and grain yield, total N uptake and N-use efficiency (NUE) at physiological maturity (148 DAS in 2011 and 153 DAS in 2012) of maize varieties from China (ZD 958 and XY 335) and US (P32D79).

Year	Variety	RDW (g plant^-1^)	R/S	Yield (t ha^-1^)	N Uptake (kg ha^-1^)	NUE (kg grain^-1^ kg N^-1^)
2011	ZD958	12.5 b	0.088ab	9.8 b	280ab	48.8ab
	XY335	9.8 b	0.072 b	8.2 c	252 b	41.2 b
	P32D79	16.9 a	0.100 a	10.9 a	315 a	54.7 a
	LSD	3.2	0.022	1.4	41	6.8
2012	ZD958	10.2 b	0.071 b	10.8 b	187 b	53.9 b
	XY335	7.4 c	0.047 c	9.6 c	199ab	47.9 c
	P32D79	15.6 a	0.087 a	12.2 a	226 a	60.9 a
	LSD	1.6	0.015	1.1	36	5.3

Field trials were performed at the Shangzhuang Experimental Station of the China Agricultural University, Beijing in 2011 and 2012.

Values in the column in each year followed by different letters had significant difference between varieties (*P*< 0.05).

## Results

### Differences in the root dry weight and root/shoot ratio of maize from China and western countries

Root dry weight increased with DAS until silking and then declined (Fig 1A). During the initial growth period (V1-V8), the difference in RDW of maize from China and western countries was not apparent (Fig 1A). After the elongation stage (V8), the difference in RDW of maize from China and western countries increased and reached the peak value when silking approached. Afterwards, RDW decreased dramatically. Compared with RDW, shoot DW increased continuously until harvest (Fig 1B). Nonlinear regression analysis indicated that maize varieties grown in western countries had larger RDW and R/S over the whole growth period and, especially, after the elongation stage (linear growth phase, V8-VT in Fig 1A and 1C). Larger R/S of maize from western countries was apparently derived from the larger RDW over the whole growth period (Fig 1).

The RDW and R/S of maize varieties grown in China and western countries were compared especially at silking when root growth was higher (Fig 1A), and at maturity when plants were harvested (Fig 2). Unpaired *t* tests showed that the average RDWs of maize grown in western countries were 25.3 g per plant at silking and 23.7 g per plant at maturity, which were significantly (*P* < 0.05) higher than those of maize grown in China at silking and maturity, which were 16.3 g and 13.2 g per plant, respectively. The average R/S of maize grown in western countries was 0.203 at silking and 0.176 at maturity, which were also significantly (*P* < 0.05) greater than those of maize grown in China at silking and maturity, which were 0.114 and 0.044, respectively (Fig 2).

**Fig 3:**
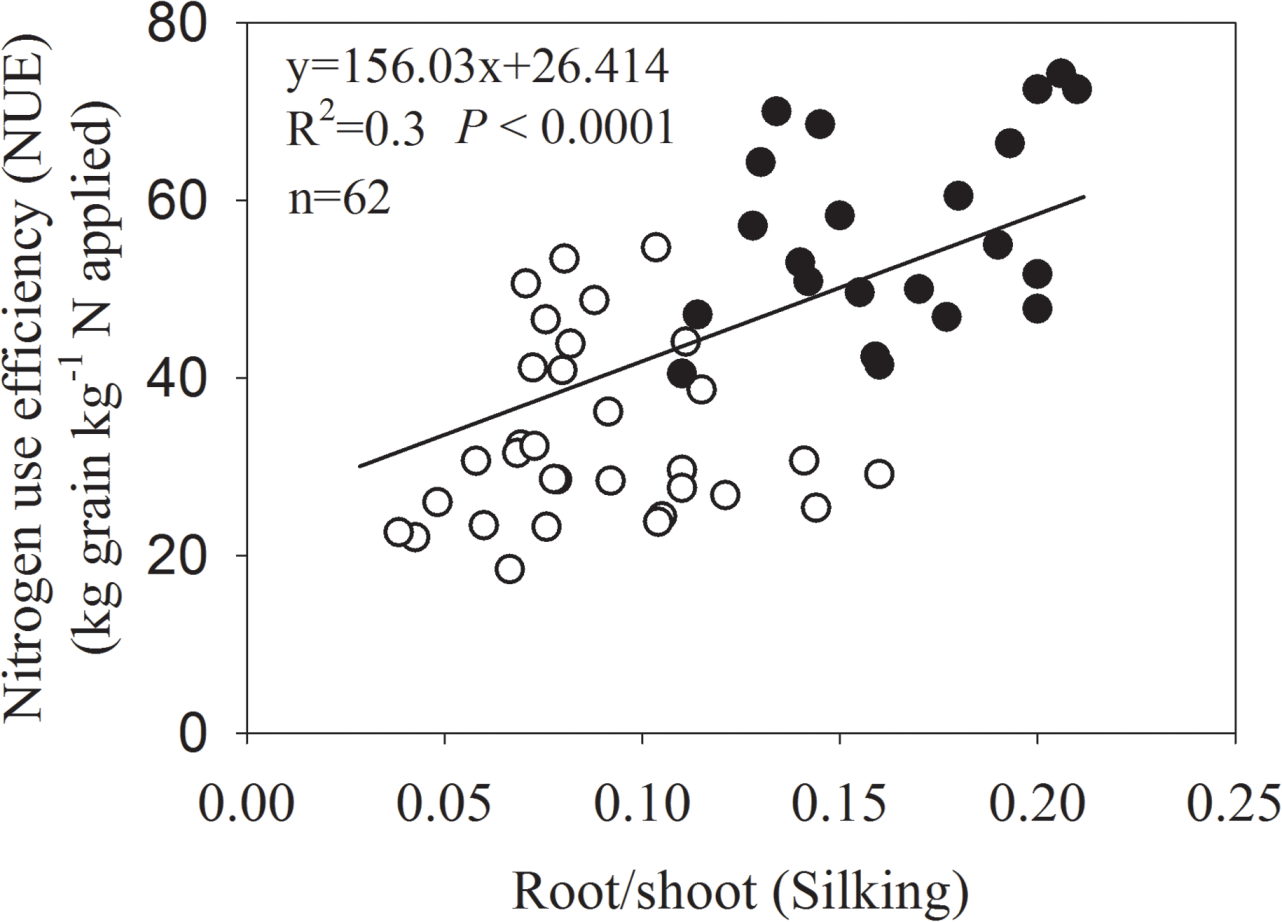
Correlation between the maize root/shoot ratio and nitrogen use efficiency (NUE) at silking. Only data published with root/shoot ratio at silking, grain yield at maturity and total N fertilizer were used in this analysis. Open circles indicate the results from Chinese farmlands and closed circles indicate data from western countries.

The correlation analysis of R/S and NUE showed that the NUE of maize was positively correlated to R/S at silking no matter where the crop was grown. The maize with smaller R/S values (Chinese varieties) had lower NUE, while the maize with larger R/S values (Western counties) had higher NUE (Fig 3).

### The influence of stress factors on the root dry weight and root/shoot ratio of maize

**Fig 4:**
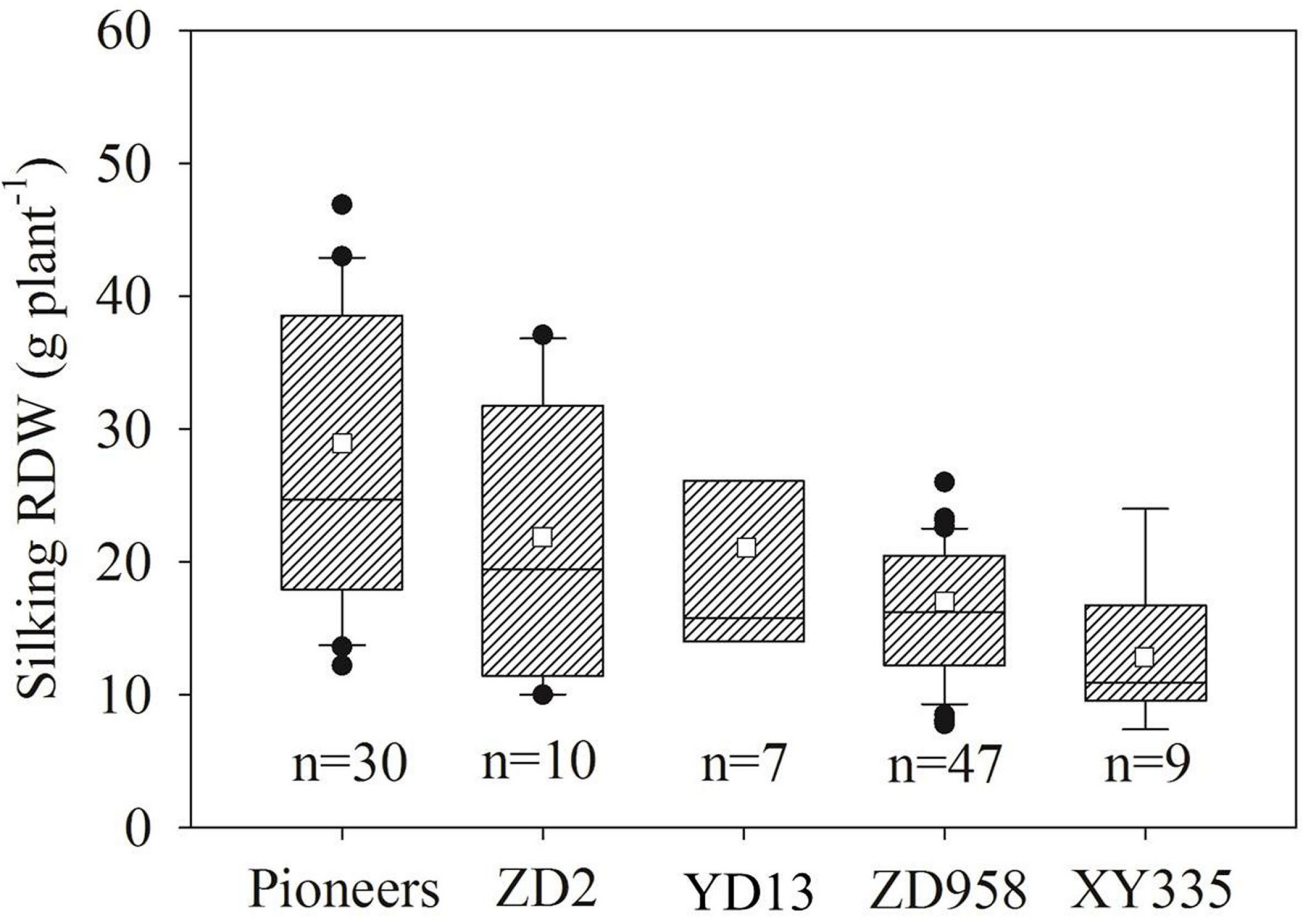
Comparisons of root dry weight at silking (silking RDW) between four dominant Chinese maize varieties and US pioneer variety. The solid line and square within the box represents the median and mean values of all data, the top and bottom edges of the box represent 75 and 25 percentiles and the top and bottom bars represent 95 and 5 percentiles of all data, respectively.

**Fig 5:**
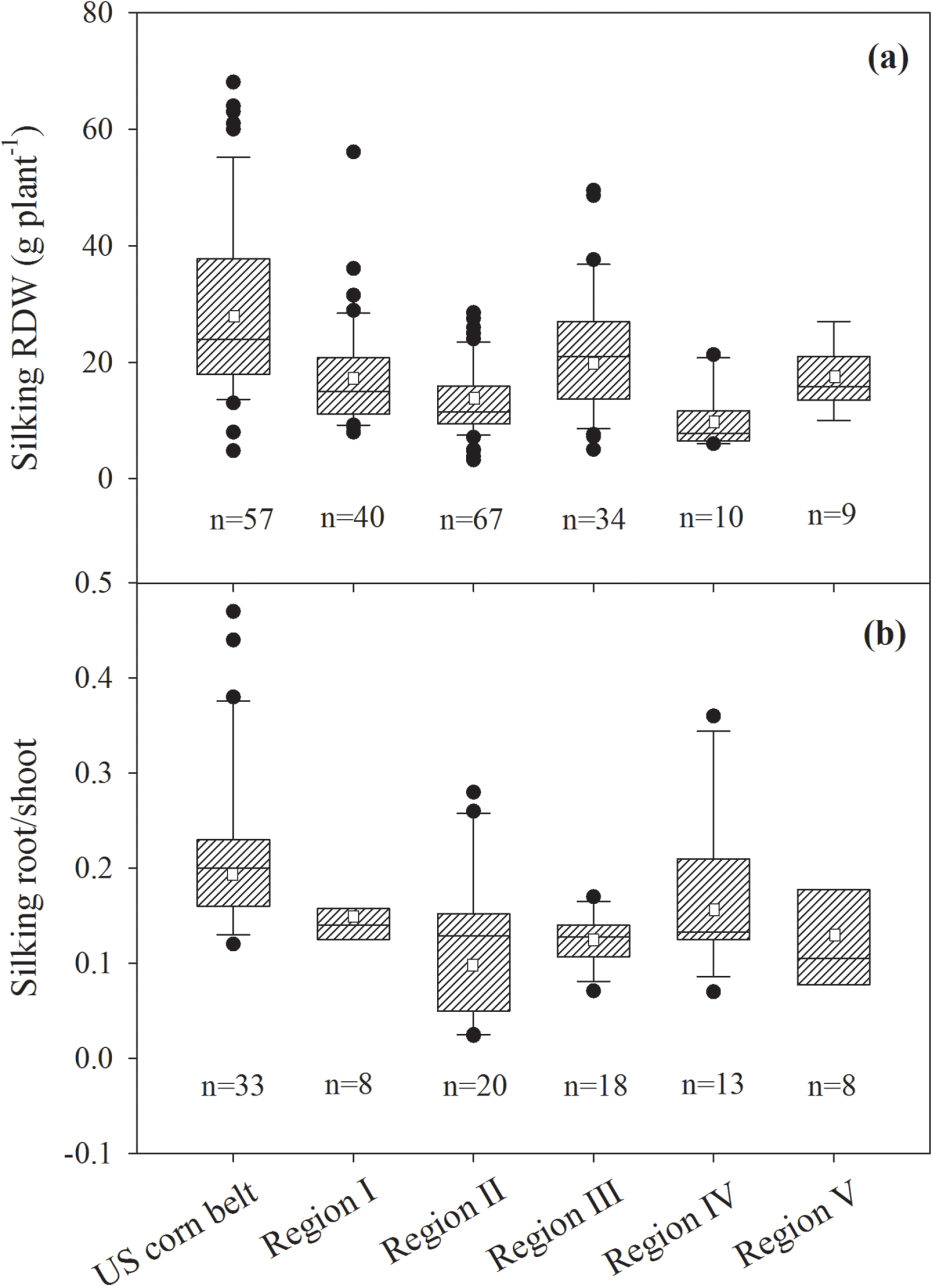
Comparisons of root dry weight at silking (silking RDW) (a) from 217 unpaired data and root/shoot ratio at silking (b) from 100 unpaired data of maize grown in US corn belt and five Chinese major maize planting regions (Regions I to V). Regions (I to V) were classified according to geographical (climatic) distinctions and soil types in China (S1 Fig). The solid line and square within the box represents the median and mean values of all data, the top and bottom edges of the box represent 75 and 25 percentiles and the top and bottom bars represent 95 and 5 percentiles of all data, respectively.

It was found that RDW at silking of maize from the US pioneer varieties were significantly larger than those of dominant maize varieties from China (Fig 4; *P*<0.05). Results showed that silking RDW and R/S of maize from the US Corn Belt were significantly larger than those of maize in all five regions in China (Fig 5; *P*<0.05).

**Fig 6:**
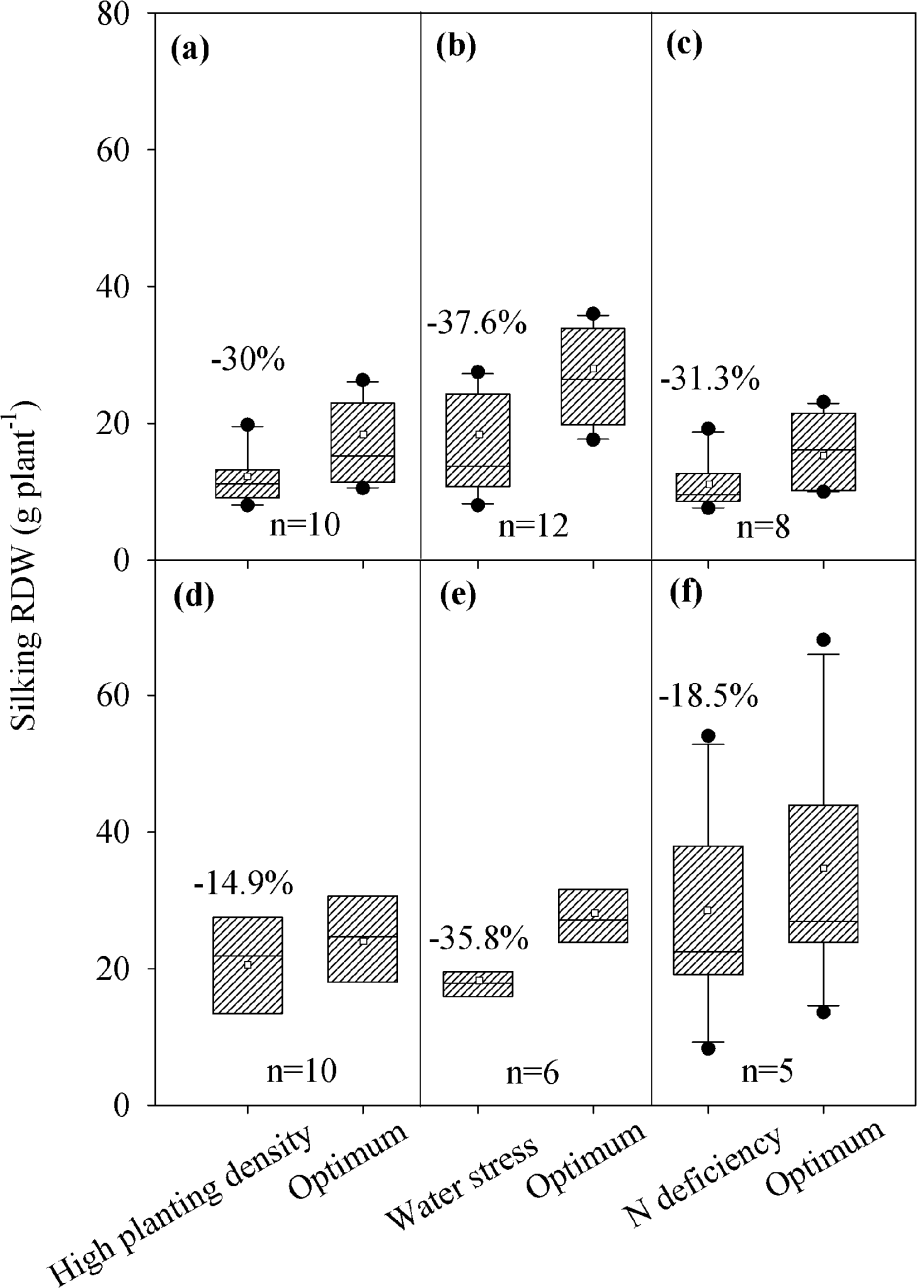
Average changes in root dry weight at silking of maize from China (a, high planting density; b, water stress; c, N deficiency) and US Corn Belt (d, high planting density; e, water stress; f, N deficiency) in response to various stresses. The data derived from individual experiments from China and US Corn Belt were used. The values above columns indicate the reduction (%) under stresses compared with the optimum conditions. The solid line and square within the box represents the median and mean values of all data, the top and bottom edges of the box represent 75 and 25 percentiles and the top and bottom bars represent 95 and 5 percentiles of all data, respectively.

Three stress factors, e.g. high plant density, drought and N deficiency, were selected to compare the responses of maize grown in China and US Corn Belt. Regardless of the overall root size, the reduction of the silking RDW of Chinese maize was more than that from the US Corn Belt, when they were subjected to these stresses, except for the drought (Fig 6).

### Field experiments with the selected varieties from China and US

US maize variety P32D79 accumulated more biomass in the root with higher R/S than two Chinese varieties at silking. The average yield and N uptake of P32D79 was 21% and 18% more than Chinese varieties, respectively. As a result, the NUE of P32D79 was significantly higher than those of two Chinese varieties in both years (Table 1). The results confirmed that the US maize variety had larger RDW and R/S at silking, take up more N and had a greater NUE than Chinese varieties (Table 1;Fig. B in S1 Information).

**Fig 7:**
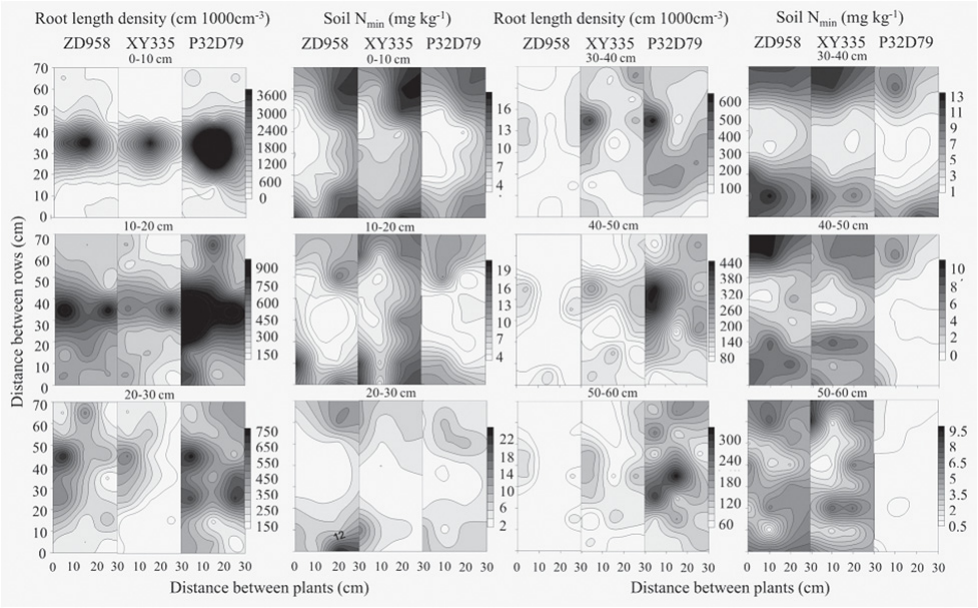
Contour maps of root length density (left) and soil mineral N (N_min_, right) concentration of three maize varieties from China (ZD 958 and XY 335) and US (P32D79) in a soil volume of 70 cm × 30 cm to a depth of 60 cm. The combined charts represented the distribution of root length density or soil N_**min**_ concentration in each soil layer, and grey scale legends indicated the relative value range. The soil samples were taken using the monolith method (Böhm, 1979) with 10 cm^3^ soil block at silking in 2011. Each soil layer contained 21 soil blocks (7 × 3, the value of each soil block was the mean of three replicates) and each root system was harvested in 126 soil blocks.

The results of the concentration of soil mineral N and the three-dimensional distributions of roots in the soil at silking showed that P32D79 had higher root length density (RLD) and lower N_min_ throughout the 0–60 cm soil profile compared with the two Chinese maize varieties (Fig 7; Fig. C in S1 Information). When comparing the two Chinese varieties ZD958 and XY335, ZD958 had higher RLD in the topsoil (<30 cm) whereas XY335 had greater RLD in the subsoil (>30 cm). This was accompanied by lower soil N_min_ in the 0–30 cm horizon and higher in the 30–60 cm horizon with ZD958 than XY335 (Fig 7; Fig. C in S1 Information). It was observed that a strong wind in 2011 resulted in lodging of XY335 with lower RLD in the 0–30 cm soil layers (Fig. D in S1 Information).

## Discussions

### The differences in the RDW and R/S of maize from China and western countries were independent of stress factors

The R/S is frequently used to estimate relative biomass allocation to the root. New maize varieties with greater RDW and R/S are likely to be more resistant to stresses factors, and, therefore, yield more grain and have a greater NUE than traditional varieties [24]. The NUE of maize was positively correlated to R/S at silking (Fig 3). The question was that what is the determining factor for the differences in RDW and R/S of maize from China and western countries at silking? RDW and R/S ratio can be a consequence of carbon allocation within plants and are influenced by environmental conditions [39–42]. Maize RDW and R/S display considerable plasticity in response to stress factors [42]. It was necessary to unravel whether stress factors contributed to major differences in RDW and R/S of maize from China and western countries.

The results demonstrated that RDW and R/S of maize at silking were greater from the US Corn Belt than those from all five regions (Figs 4 and 5) in China, including the maize in Region I (northeast China Plain) where the soil and climatic conditions are almost similar to those in the US Corn Belt [43, 44]. Therefore, the larger RDW and R/S of the maize from western countries than those from China are independent of the differences in the growing environments. Greater RDW and R/S in maize from western countries are largely due to differences in genetic composition (Figs 4 and 5). This conclusion was also supported by the present field experiments with two Chinese and one American varieties under the same conditions (Table 1; Fig. B in S1 Information). Shandong province (Region V) is an intensive agricultural and economically developed region in China, with large amount of chemical fertilizer input in agricultural production. The maize in this region had higher grain yield [45, 46]; however, the average R/S of maize at silking was the smallest among the five regions (Fig 5B), because the over-use of chemical N fertilizer probably inhibits root growth [47].

Different stresses influence maize RDW and R/S differently. Nutrient deficiency generally results in an increase in the overall maize R/S, while water stress, high plant density, shading, and soil compaction lead to a decrease in maize R/S [42]. Comparative analyses indicated that the reduction in the RDW of differently stressed Chinese maize at silking was more than that of the maize from the US Corn Belt (Fig 6), the values of the RDW reductions were similar to the reported average maize RDW reduction of 30.1% in response to high population, 44.2% in response to drought, and markedly lower than the reduction of 6.5% in response to N deficiency reviewed by Amos and Walters 2006 [42]. The maize from the US Corn Belt had less reduction in RDW at silking under high planting density and N deficiency, implying that maize bred in the US is more stress tolerance than those bred in China. Genetic improvement of enhanced maize grain yield is largely dependent on the improved stress tolerance in the US maize breeding practices [25, 48, 49]. In China, however, recently released varieties do not show improvement in tolerance to high plant density [29, 50–53].

### Ideal root distribution is beneficial for higher grain yield and nitrogen-use efficiency

Field experiments with two widely used maize varieties bred in China (ZD958 and XY335) and one bred in the US (P32D79) under the same growth conditions confirmed that the P32D79 with larger RDW and R/S at silking acquired more N and had a greater NUE (Table 1; Fig. B in S1 Information). The results validated the relationships between R/S at silking and maize NUE (Fig 3).

Efficient N uptake depends not only on root size but also on the root distribution in the soil profile [16, 17, 54]. Detailed studies on the three-dimensional distributions of roots in the soil and the concentration of soil mineral N at silking revealed a negative correlation (0.5<R^2^<0.7) between RLD and soil N_min_. Greater RLD in 0–60 cm soil profile resulted in P32D79 acquiring more N, leaving less N_min_ in the soil, and having a higher NUE than the two Chinese varieties (Table 1; Fig 7; Fig. B and C in S1 Information). Maize varieties with greater RLD in the top soil depleted more soil mineral N and reduced the movement of water and nitrate-N towards deeper soil layers [16, 17, 55]. In addition, maize varieties with large RDW and RLD in the top soil ensure that plants remain upright and do not suffer from the dramatic yield loss associated with lodging [56] (Fig. D in S1 Information). Varieties with greater RLD in the subsoil can reduce N leaching by absorbing N. Additionally, deep roots enable plants to access water in deeper soil horizons thereby reducing the risk of drought stress [54, 57, 58].

An ideal root architecture for N uptake includes large RLD in both the top soil and subsoil [16, 17, 54]. This result suggests that high RLD is beneficial for the better uptake of nutrients, such as phosphorus [59] and N. It is possible that Chinese farmers might have over-applied N fertilizers to compensate for the small root systems and low tolerance to the stresses. Unfortunately, excess N can inhibit root growth, especially at early growth stages of maize plants [18] which can reduce NUE with increased environmental consequences of groundwater N contamination and greenhouse gas (N_2_O) emissions.

Optimal management of mineral nutrient inputs has become one of the most urgent requirements for sustaining intensive agriculture in China [16, 17, 54]. Most reports suggest that the excessive use of N fertilizers leads to low NUE in intensive Chinese agriculture and have serious environmental consequences [11, 13, 18, 60]. China is taking steps towards increasing grain yield and NUE by integrated agronomic and nutrient management approaches [13, 60–62]. However, there are still large gap in NUE of maize production between China and western countries [13, 14]. Our study identified target root traits and tolerance to stresses that will improve NUE in maize production both in China and elsewhere in the world. In the long run, breeding crops with favorable RDW, large R/S and better root system architecture could make significant contribution to increasing NUE, while reducing the environmental risks of N fertilization. These root traits will enable crop breeders to select and breed appropriate genotypes to increase NUE in Chinese cropping systems [22, 23, 63, 64].

## Supporting Information

S1 Information Detailed materials and methods. (DOC)
