# Supplementary material for: A Large and Deep Root System Underlies High Nitrogen-Use Efficiency in Maize Production
Source: PLoS One. 2015 May 15;10(5):e0126293. doi: 10.1371/journal.pone.0126293 (PMC4433229; doi:10.1371/journal.pone.0126293)
Supplement: S1 Information — (DOC) [file pone.0126293.s001.doc]

**S1 Supporting Information**

**Data analysis**

**Coverage of data collected in China**

Studies reported in the literature were carried out in 13 major maize production provinces in five regions in China: north China, northeast China, southwest China, northwest China and east China. The geographical distribution of the data is shown in Figure A. The data are derived from all major maize production areas in China and provide a complete survey of maize growth conditions in China (Figure E). However, data had large variations due to differences in climate and soil conditions beteween different regions. Among 709 values for RDW reported in Figure 1, 31.2% were collected in northeast and east China and had large RDW, 43.6% were collected in north China and had small RDW, and 25.2% were collected in southwest and northwest China and had medium RDW plant^-1^. Among the 390 data for R/S in the same figure, 22.4% were collected in northeast and east China and had large R/S ratios, 40.6% were collected in north China and had small R/S ratios, and 37% were collected in southwest and northwest China and had medium R/S ratios (Figure Ea, b).

Among the 178 values for RDW at silking in Fig. 2, 26.2% were collected in northeast and east China and had large RDW (> 17.4 g plant^-1^), 44.9% were collected in north China and had small RDW (< 10.8 g plant^-1^) and 28.9% were collected in southwest and northwest China and had medium RDW (10.8 - 17.4 g plant^-1^). Among the 74 values for R/S at silking, 19.1% were collected in northeast and east China and had large R/S (> 0.125), 37.1% were collected in north China and had small R/S (< 0.074); and 43.8% were collected in southwest and northwest China and had medium R/S (0.074 - 0.125) (Figure Ec, d).

**Variation in the RDW in field studies performed in different eras in China**

In comparison with the large variations of silking RDW of maize grown in different regions (Fig. 5), no significant difference in the RDW of maize grown in trials pre-1990s and post-1990s were observed either at silking (18.7 g plant^-1^ vs. 17.0 g plant^-1^) or at physiological maturity (13.0 g plant^-1^ vs. 14.3 g plant^-1^) (Figure F). Therefore, the era of field trials had less influence on RDW than the location of the trial.

**Variations in sampling methods and soil fertility**

Variation in the data could be resulted from differences in root sampling methods and post-sampling procedures such as root washing. The common methods [1] of root sampling in the field include soil coring [2-4], whole root excavation [5, 6], stratified removal of soil horizons [4-7], and the monolith method [8-10]. For the soil core method, the number of cores extracted and the pattern of core extraction can lead to differences in the RDW recorded. Root washing after root sampling can also lead to root losses and a smaller RDW [11].

Among the 66 studies undertaken in China, 30.4%, 12.4%, 41.1% and 1.8% of harvested roots using whole root excavation, the soil cores method, the stratified method and the monolith method, respectively. The other 14.3% did not describe how root systems were sampled in detail (Figure Ga).

Soil fertility affects root growth and development [12-14]. According to region-specific soil type and soil developmental process (Second National Soil Survey in China, 1979-1985), soil fertility where the field experiments were performed falls into three categories modified from China 2^nd^ national soil survey: high (SOM>40 g kg^-1^, total N>2 g kg^-1^, total P>1 g kg^-1^, total K>25 g kg^-1^); medium (SOM 10-30 g kg^-1^, total N 0.75-1.5 g kg^-1^, total P 0.4-0.8 g kg^-1^, total K 10-20 g kg^-1^); and low (SOM<6 g kg^-1^, total N<0.5 g kg^-1^, total P<0.2 g kg^-1^, total K<5 g kg^-1^) fertility. Approximately 37.5% of the data were obtained from experiments on high fertility soils, including region-specific soil types such as the black soil, lime chernozems, meadow chernozemic soil, ustic isohumisol, purplish soil, dark brown and brown earth soil, and soil types undergoing special developmental processes, such as the alluvial soil, fluvo-aquic soil, and Lou soil. 41.2% of the data were obtained from experiments on low fertility soils, mainly in the sandy loam, clay loam, and lime concretion black soils. The other 21.4% were from experiments performed on medium fertility soils such as the brown earth soil, serozem soil, red earth soil and yellow earth soil (Figure Gb). Therefore, the analysis reported in this paper covered many soil types and soil fertility variations in China.

**Correlation between the R/S ratio and N fertilizer use efficiency**

Maize has maximum RDW at silking [7]. Only articles reporting R/S dry weight ratio at silking, grain yield at maturity, and total N fertilizer input were used to determine the correlation between R/S and nitrogen use efficiency (NUE). Correlation coefficients were obtained using Sigmaplot 12.0 (Systat Software Inc.). Signiﬁcant positive correlation between the R/S at silking and NUE, which reflects yield production per-unit-N fertilizer application, is plotted in Fig. 3 in the main text (R^2^ = 0.2973; n = 62).

**References**

1. Amos B, Walters DT (2006) Maize root biomass and net rhizodeposited carbon: An analysis of the literature. Soil Sci Soc Am J 70: 1489–1503.
2. Kaspar TC, Brown HJ, Kassmeyer EM (1991) Corn root distribution as affected by tillage, wheel traffic, and fertilizer placement. Soil Sci Soc Am J 55: 1390–1394.
3. Kovar JL, Barber SA, Kladivko EJ, Griffith DR (1992) Characterization of soil temperature, water content, and maize root distribution in two tillage systems. Soil Tillage Res 24: 11–27.
4. Peng YF, Yu P, Li XX, Li CJ (2013) Determination of the critical soil mineral nitrogen concentration for maximizing maize grain yield. Plant Soil 372: 41–51.
5. Piper EL, Weiss A (1993) Defoliation during vegetative growth of corn: The shoot: root ratio and yield implications. Field Crops Res 31: 145–153.
6. Ma BL, Dwyer LM, Costa C (2003) Row spacing and fertilizer nitrogen effects on plant growth and grain yield of maize. Can J Plant Sci 83: 241–247.
7. Peng YF, Li XX, Li CJ (2012) Temporal and spatial profiling of root growth revealed novel response of maize roots under various nitrogen supplies in the field. PLoS ONE 7: 1–11.
8. Böhm W (1979) Methods of Studying Root Systems. Berlin: Springer-Berlag Press.
9. Kuchenbuch RO, Gerke HH, Buczko U (2009) Spatial distribution of maize roots by complete 3D soil monolith sampling. Plant Soil 315: 297–314.
10. Peng YF, Niu JF, Peng ZP, Zhang FS, Li CJ (2010) Shoot growth potential drives N uptake in maize plants and correlates with root growth in the soil. Field Crops Res 115: 85–93.
11. Oliveira MRG et al. (2000) In: Smit AL, editor. Root methods: A handbook. (eds, pp. 175–210, Berlin: Springer-Verlag press.
12. Anderson EL (1987). Corn root growth and distribution as influenced by tillage and nitrogen fertilizer. Agro J 79: 544–599.
13. Laboski CAM (1998) Soil strength and water content influences on corn root distribution in a sandy soil. Plant Soil 203: 239–247.
14. Goodman AM, Eends AP (1999) The effects of soil bulk density on the morphology and anchorage mechanics of the root systems of sunflower and maize. Ann Bot 83: 293–302.

**Figure A. Five major maize production areas in China**: northeast China (Heilongjiang, Jilin and Liaoning provinces), north China (Beijing, Hebei, Henan and Shanxi provinces), northwest China (Xinjiang, Inner Mongolia, Shaanxi and Gansu provinces), southwest China (Chongqing, Sichuan and Guizhou provinces), and east China (mainly Shandong province). Dots with different color indicate the frequency of individual trials (66 field experiments) in the five major maize production regions.


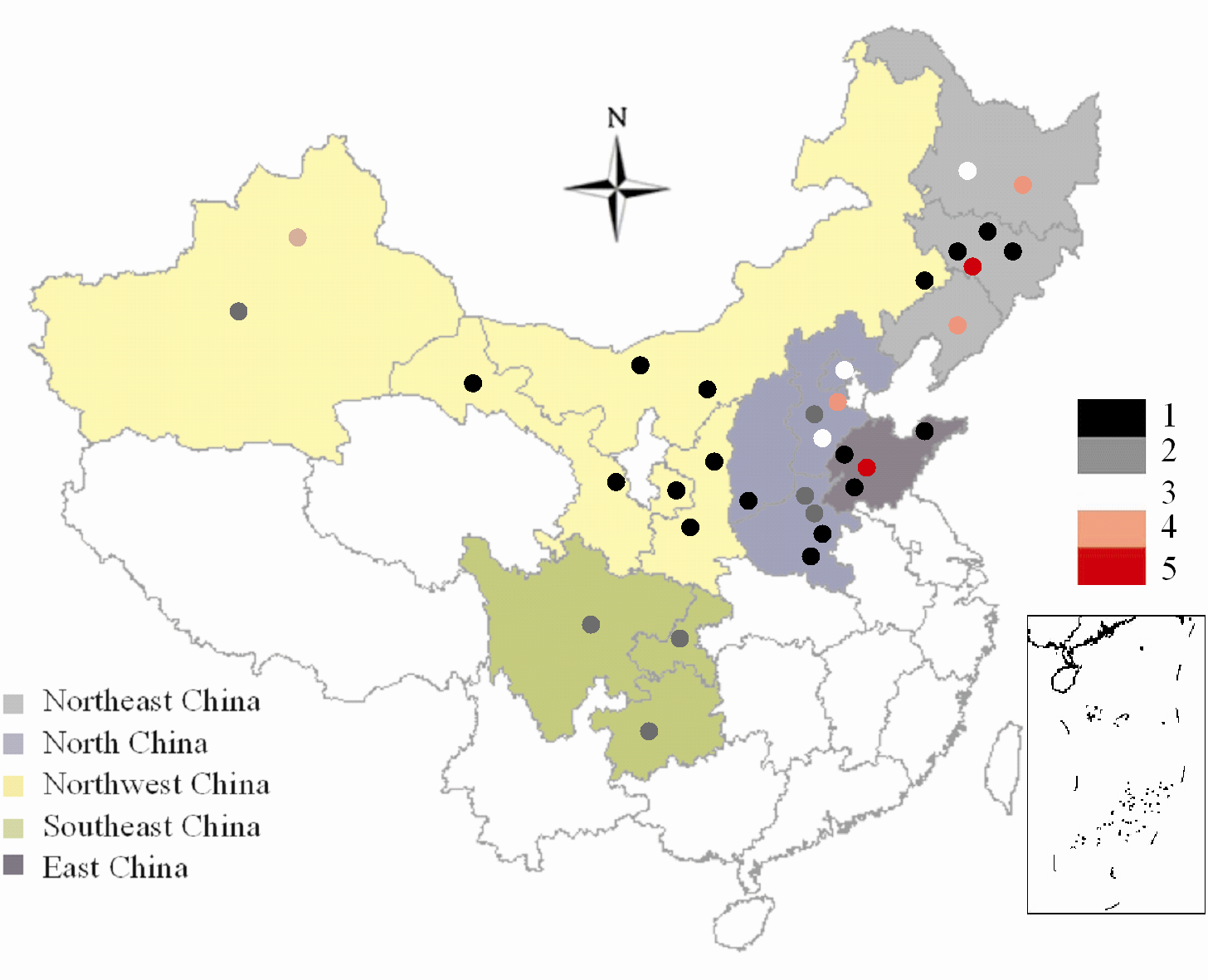


**Figure B. Roots of maize varieties from China (ZD 958 and XY 335) and the US (P32D79) excavated at silking in 2011.**

**
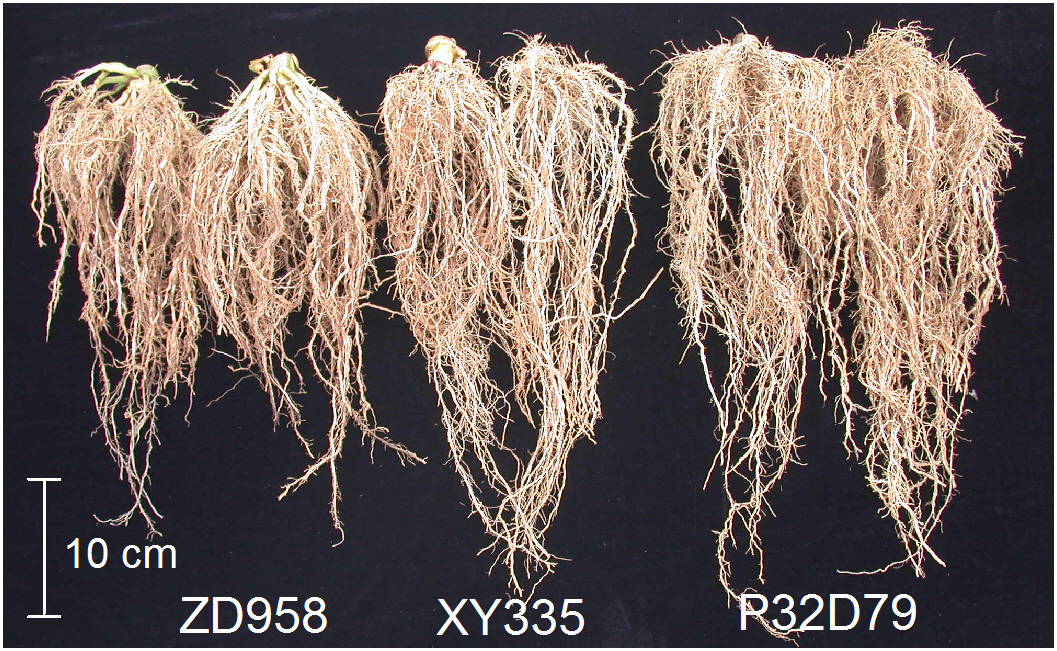
**

**Figure C. Contour maps of root length density (left) and soil mineral N (N_min_) concentration (right) of maize varieties from China (ZD958 and XY335) and the USA (P32D79) at silking in 2012.** The charts represent the distribution of root length density or soil N_min_ concentration in each soil horizon (10 cm each). The grey scale legend indicates the relative value range. The soil samples were taken using the monolith method (Böhm, 1979) with 10 cm^3^ soil block. Each soil layer contained 15 soil blocks (5 × 3, the value of each soil block was the mean of three replicates) and each root sample was harvested in 90 soil blocks. The soil volume was 50 cm×30 cm×60 cm (length×width×depth).


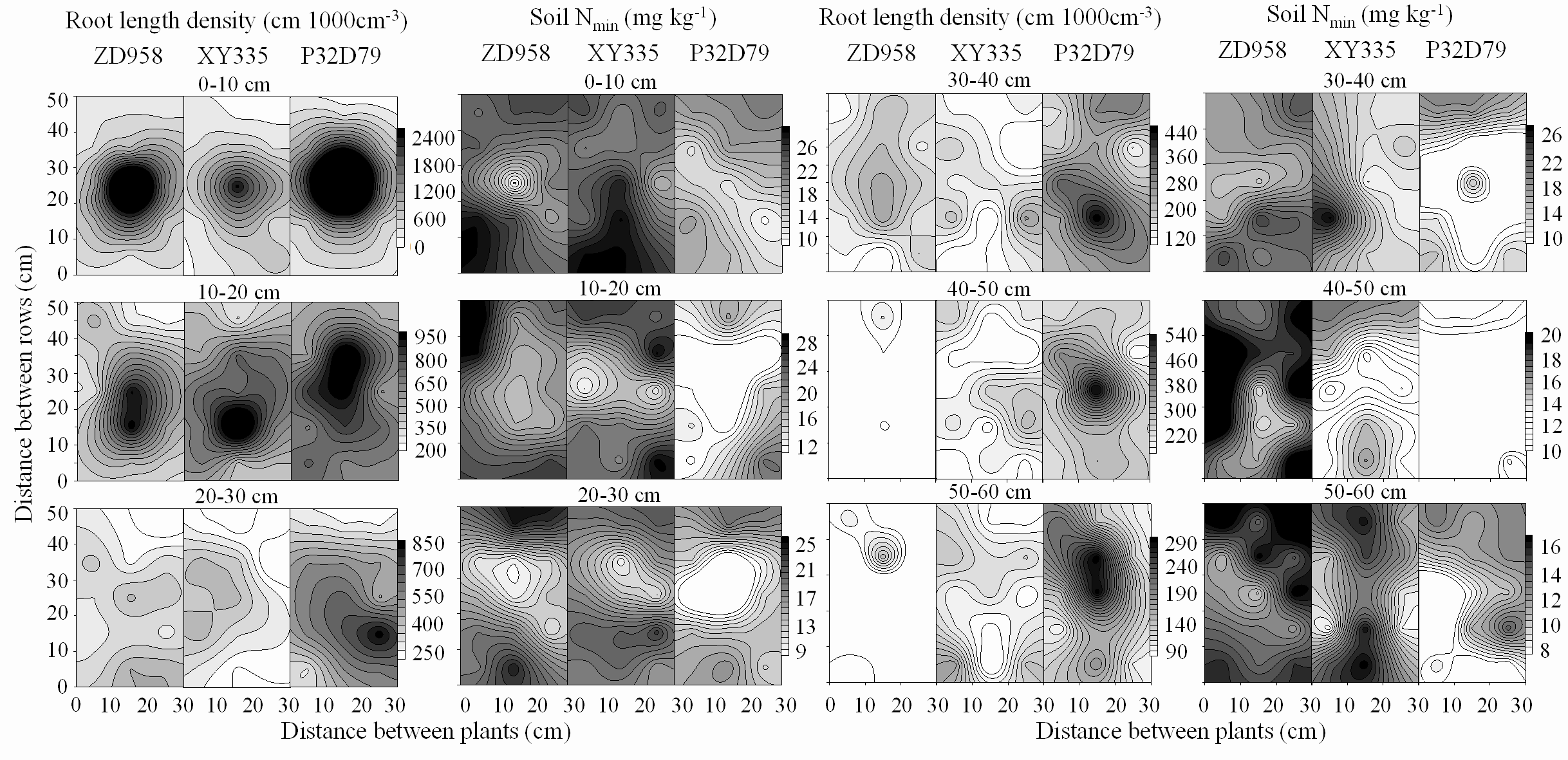


**Figure D. Maize plants after a strong wind. Images were taken two weeks after silking in 2011**. Left: ZD 958; Middle: XY 335; Right: P32D79.


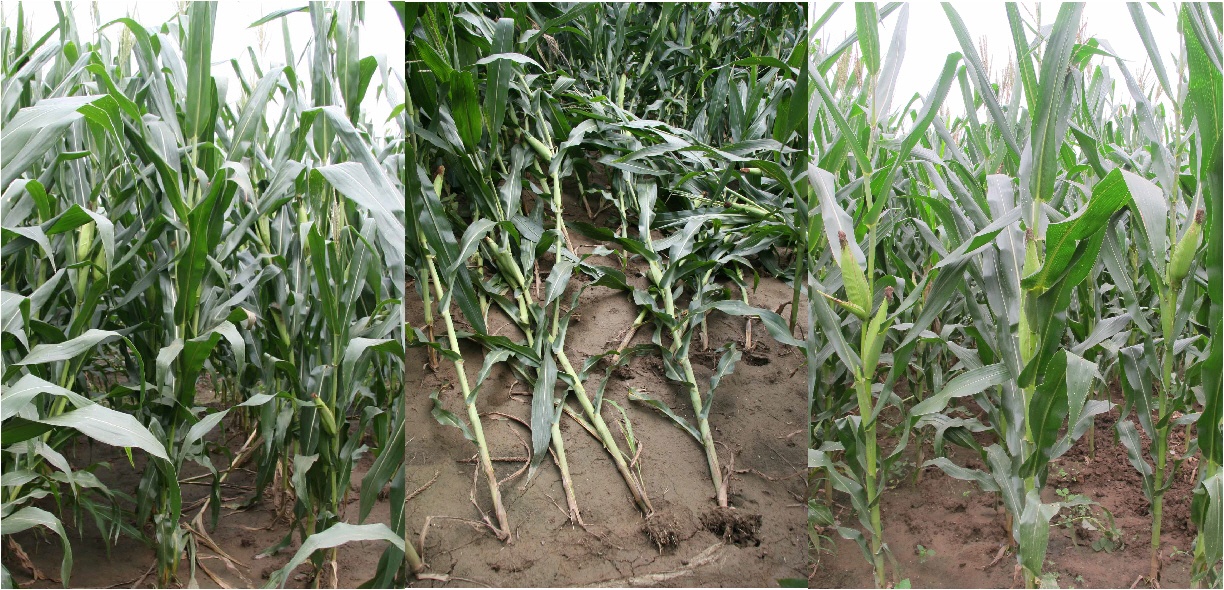


**Figure E. Regional distribution of the 709 and 390 data points used to generate the time course of changes in RDW (a) and R/S (b) in maize grown in China (Fig. 1), and 178 and 74 data points used to analyze RDW (c) and R/S (d) at silking in maize grown in China (Fig. 2)**.

**Figure F. Comparison of RDW of maize grown in the field in different eras (1970-1990, 1990-present) in China at silking and maturity.** The varieties were separated on date of release irrespective of which year the data were published. The results in two upper panels were presented as g plant^-1^ and those in two lower panels as t ha^-1^.


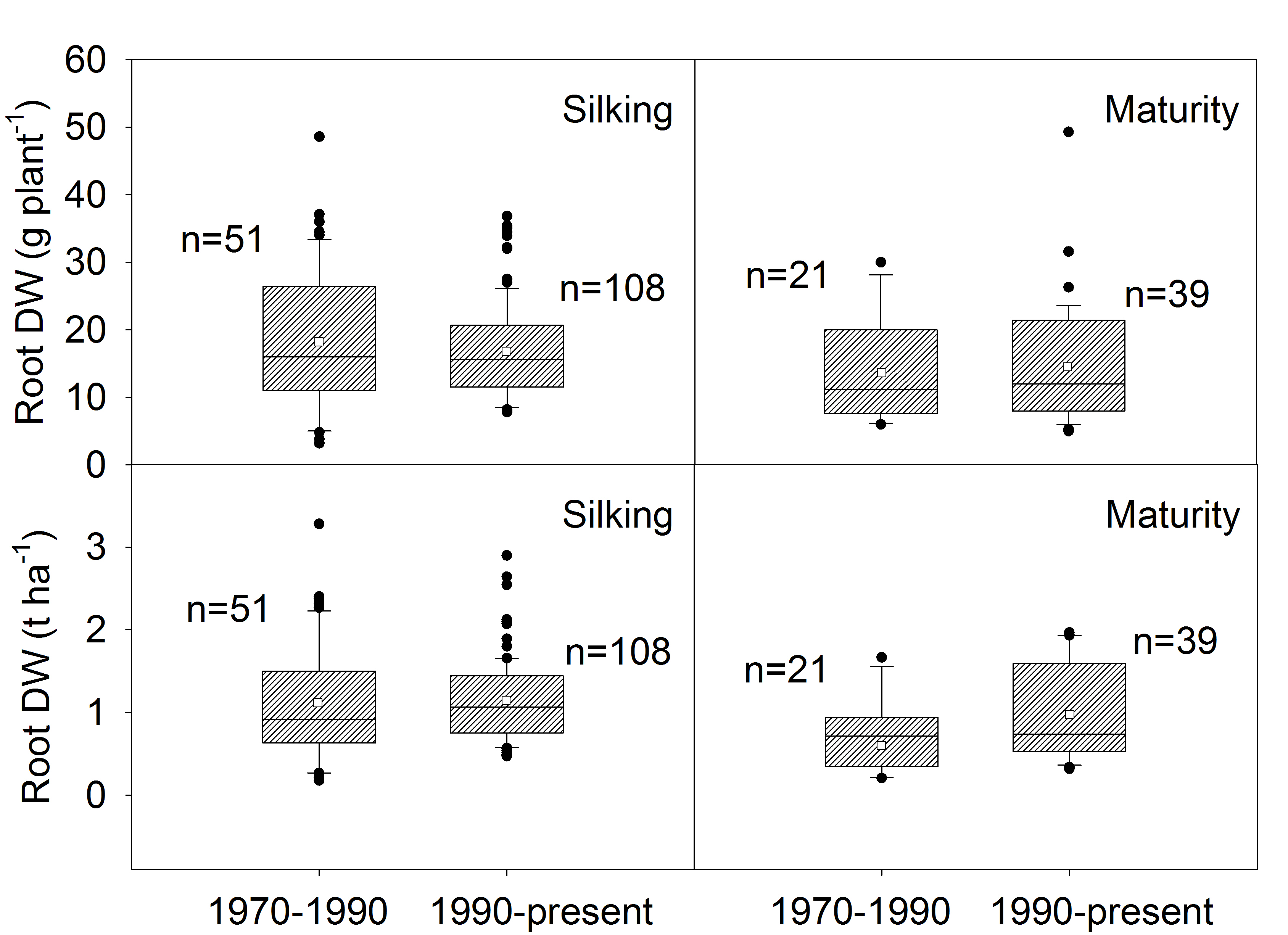


**Figure G. Root sampling methods (a) and soil fertility (b) for the studies performed in China.**

**Table A. List of 106 field studies reporting maize root dry weight and root/shoot dry weight ratio published in 53 (35 in Chinese and 18 in English) journals since 1959.** There were 66 studies performed in China and 40 in the America and Europe.

| NO. | Language | Type | Author list | Journal | Year Published,  Volume and Pages |
| --- | --- | --- | --- | --- | --- |
| 1 | C | [J] | Chen SQ, Xu HT, Duan CP. | Journal of Hebei Agricultural Science. | 2011. 15: 62-64, 95. |
| 2 | C | [J] | Chen YL, Wu QP, Chen XC, Chen FJ, Zhang YJ, Li Q, Yuan LX, Mi GH. | Plant Nutrition and Fertilizer Science. | 2012. 18: 52-59. |
| 3 | C | [J] | Chun L, Chen FJ, Zhang FS, Mi GH. | Plant Nutrition and Fertilizer Science. | 2005. 11: 615-619. |
| 4 | C | [J] | E YJ, Dai JY, Gu WL. | Acta Agrinomica Sinica. | 1988. 14: 149-154 |
| 5 | C | [J] | Fan XY, Yang HS, Gao JL, Zhang RF, Wang ZG, Zhang YQ. | Plant Nutrition and Fertilizer Science. | 2012. 18: 562-570. |
| 6 | C | [J] | Gu WL, E YJ, Dai JY. | Journal of Shenyang Agricultural University. | 1988. 19: 1-6. |
| 7 | C | [J] | Huang RD, Ma HT. | Tillage and Cultivation. | 1993. 4: 21-23. |
| 8 | C | [J] | Kong DY, Zhi H, Zhang FQ, Zhang LX, Han J. | Chinese Journal of Agrometeorology. | 2008. 29: 67-70. |
| 9 | C | [J] | Lan HL, Dong ZQ, Pei ZC, Xu TJ, Xie ZX. | Journal of Maize Science. | 2011. 19: 62-69. |
| 10 | C | [J] | Li CH, Zhou SL, Fan YT, Zhao QH, Li JZ. | Journal of Henan Agricultural University. | 1996. 30: 249-253. |
| 11 | C | [J] | Li L, Xu YL, Guo PL. | Journal of Maize Science. | 1993. 1: 57-60. |
| 12 | C | [J] | Li SK, Liu JD, Luo ZG, Zhang WF, Wei BJ. | Journal of Shihezi University. | 1993. 4: 1-4. |
| 13 | C | [J] | Li SK, Liu JD, Zhang WF, Wei BJ, Yang G, Zhao H. | Journal of Maize Science. | 1993. 1: 43-49. |
| 14 | C | [J] | Li SK, Tu HY, Zhang WF, Yang G. | Journal of Xinjiang Agricultural Science. | 1992. 3: 99-103. |
| 15 | C | [J] | Li SK, Tu HY, Zhang WF. | Journal of Shihezi University. | 1992. 4: 1-5. |
| 16 | C | [J] | Li SK, Zhang WF, Wang ZY, Hu XT, Wei BJ, Yang G. | Journal of Shihezi University. | 1998. (supp.) :81-88. |
| 17 | C | [J] | Li YY, Liu WZ. | Chinese Journal of Eco-Agriculture. | 2001. 9: 13-15. |
| 18 | C | [J] | Liang JB, Liu JH, Yang T. | Journal of Anhui Agricultural Science. | 2006. 34: 2353-2354. |
| 19 | C | [J] | Liang YC, Yu GX, Yang DR, Liu QJ. | Agriculture Research in the Arid Areas. | 1990. 1: 27-32. |
| 20 | C | [J] | Liu CW, Zhang EH, Xie RZ, Liu WR, Li SK. | Chinese Journal of Eco-Agriculture. | 2012. 20: 203-209. |
| 21 | C | [J] | Liu JB, Wang XL, Zhang SQ, Zhang RH, Xue JQ. | Bulletin of Soil and Water Conservation. | 2011. 31: 32-36, 41. |
| 22 | E | [J] | Liu JX, Chen FJ, Olokhnuud CL, Glass ADM, Tong YP, Zhang FS, Mi GH. | Journal of Plant Nutrition and Soil Science. | 2009. 172: 230-236. |
| 23 | C | [J] | Liu PL, Lin Q, Sui FG, Sun ZQ. | Journal of Maize Science. | 1994. 2: 59-63. |
| 24 | C | [J] | Liu SQ, Song FB, Wang Y. | Journal of Jilin Agricultural University. | 2007. 29: 1-6. |
| 25 | C | [J] | Liu ZD, Xiao JF, Yu JH, Nan JQ, Liu ZG. | Journal of Irrigation and Drainage. | 2011. 30: 44-47. |
| 26 | C | [J] | Liu ZW, Xie RZ, Zhang EH, Liu WR, Li SK. | Journal of Maize Science. | 2009. 17: 120-123. |
| 27 | C | [J] | Lu HD, Xue JQ, Ma GS, Hao YC, Zhang RH, Ma XF. | Chinese Journal of Applied Ecology. | 2010. 21: 895-900. |
| 28 | E | [J] | Ning P, Liao CS, Li S, Yu P, Zhang Y, Li XX, Li CJ. | Field Crops Research. | 2012. 130: 38-45. |
| 29 | E | [J] | Niu JF, Peng YF, Li CJ, Zhang FS. | Journal of Plant Nutrition and Soil Science. | 2010. 173: 306-314. |
| 30 | E | [J] | Peng YF, Niu JF, Peng ZP, Zhang FS, Li CJ. | Field Crops Research. | 2010. 115: 85-93. |
| 31 | C | [J] | Peng ZP, Zhang JT, Yuan S, Wang YQ, Liu HL, Xue SC. | Plant Nutrition and Fertilizer Science. | 2009. 15: 793-798. |
| 32 | C | [J] | Qi WZ, Liu HH, Li G, Shao LJ, Wang FF, Liu P, Dong ST, Zhang JW, Zhao B. | Plant Nutrition and Fertilizer Science. | 2012. 18: 69-76. |
| 33 | C | [J] | Ren SX, Zhao HR, Huo ZG, Wang SY. | Journal of Soil and Water Conservation. | 2004. 18: 161-165. |
| 34 | C | [J] | Shen XS, Li JC, Qu HJ, Wei FZ, Zhang Y, Wu WM. | Scientia Agricultura Sinica. | 2011. 44: 2005-2012. |
| 35 | C | [J] | Song FB, Xu C, Yan XL, Dai JY. | Journal of Jilin Agricultural University. | 1997. 19: 18-22. |
| 36 | C | [J] | Song R, Wu CS, Mou JM, Xu KZ. | Journal of Jilin Agricultural University. | 2000. 22: 73-75, 80. |
| 37 | C | [J] | Su CH, Weng HY, Ren PH. | Journal of Shanxi Agricultural Science. | 1996. 24: 42-46. |
| 38 | C | [J] | Sun QQ, Hu CH, Dong ST, Wang KJ. | Acta Agrinomica Sinica. | 2003. 29: 641-645. |
| 39 | C | [J] | Wang JH, Hu JL, Lin XG, Dai J, Wang JT, Cui XC, Qin SW. | Acta Pedologica Sinica. | 2011. 48: 766-772. |
| 40 | C | [J] | Wang KJ, Zheng HJ, Liu KC, Zhang JW, Dong ST, Hu CH. | Acta Phytoecologica Sinica | 2001. 25: 472-475. |
| 41 | C | [J] | Wang M, Feng DY, Yi Y, Qian XG, Liang YS. | Tillage and Cultivation. | 2007. 4: 14-16. |
| 42 | C | [J] | Wang MX, Tao YX. | Acta Agriculturae Boreali-Sinica. | 2011. 10: 13-16. |
| 43 | C | [J] | Wang QX, Wang P, Yang XY, Zhai ZX, Wang XL, Shen LX. | Scientia Agricultura Sinica | 2003. 36:1469-1475. |
| 44 | C | [J] | Wang S, Chen JZ, Luo Y. | Plant Nutrition and Fertilizer Science. | 2008. 14: 646-651. |
| 45 | C | [J] | Wu RJ, Zheng YF, Wang CH, Hu ZH. | Ecology and Environment. | 2007. 16: 323-326. |
| 46 | C | [J] | Xu CZ, Sun XM. | Journal of Maize Science. | 2008. 16: 101-103. |
| 47 | C | [J] | Xue JF, Wang JK, Li SY, Zhu FC, Chen SQ. | Journal of Maize Science. | 2006. 14: 66-70. |
| 48 | C | [J] | Yan Y, Liao CS, Zhang FS, Li CJ. | Plant Nutrition and Fertilizer Science. | 2010. 16: 257-265. |
| 49 | C | [J] | Yang QH, Gao EM, Ma XM, Wang HC, Sun ZA, Yin F, Liu B, Ren J, Wang Q. | Acta Agriculturae Boreali-Sinica. | 2000. 15: 88-93. |
| 50 | C | [J] | Yang QH, Gao EM, Ma XM. | Chinese Journal of Ecology. | 2000. 19: 28-31. |
| 51 | C | [J] | Yang QH, Gao EM, Ma XM. | Chinese Journal of Soil Science. | 2001. 32: 238-240. |
| 52 | C | [J] | Yang WB, Bai DC, Dong XC, Tian YD. | Acta Agriculturae Boreali-Sinica. | 1987. 2: 44-51. |
| 53 | C | [J] | Yi ZX, Wang P, Tu NM. | Plant Nutrition and Fertilizer Science. | 2009. 5: 91-98. |
| 54 | C | [J] | Yin CX, Li GH, Zhang SX, Xie JG, Wang XF, Zhang K. | Journal of Maize Science. | 2011. 19: 112-115. |
| 55 | C | [J] | Yong TW, Yang WJ, Xiang DB, Chen XR. | Chinese Journal of Applied Ecology. | 2012. 23: 125-132. |
| 56 | C | [J] | Yuan JC, Yang WY, Ni S. | Journal of Sichuan Agricultural University. | 1996. 14: 183-186. |
| 57 | C | [J] | Yuan Y, Zhang YM, Zhao J, Guo L, Zhang FL. | Journal of Maize Science. | 2011. 19: 110-112, 117 |
| 58 | C | [J] | Zhan J, Zhang YM, Niu XK, Liu X, Li SK, Zhang FL. | Acta Agriculturae Boreali-Sinica. | 2011. 26 (suppl): 99-103. |
| 59 | C | [J] | Zhang EH, Huang GB. | Chinese Journal of Applied Ecology. | 2003. 14: 1301-1304. |
| 60 | C | [J] | Zhang FL, Niu XK, Zhang YM, Li SK, Xie RZ, Liu X, Xiu WW. | Acta Agriculturae Boreali-Sinica. | 2012. 27:146-151. |
| 61 | C | [J] | Zhang GG, Hou TR, Wu MQ, Li XJ, Cao XC, Zhang X, Yu JQ, Li WJ, Lian GY, Liu ZH, Zhang GZ, Zou SF. | Rain Fed Crops. | 2003. 23: 212-213. |
| 62 | C | [J] | Zhang LJ, Zhang EH. | Chinese Agricultural Science Bulletin. | 2006. 12: 133-138. |
| 63 | C | [J] | Zhang YQ, Yang HS, Gao JL, Zhang RF, Wang ZG, Xu SJ, Fan XY, Bi WB. | Acta Agrinomica Sinica. | 2011. 37: 735-743. |
| 64 | C | [J] | Zhao BQ, Zhang FS, Li ZJ, Li FC, Shi CY, Zhang J, Zhang XC, Shen JX, Pan HJ, Zhao JM. | Plant Nutrition and Fertilizer Science. | 2003. 9: 81-86. |
| 65 | C | [J] | Zhao BQ, Zhang FS, Li ZJ, Li FC, Zhang XC, Shen JX, Pan HJ, Zhao JM, Yin YB, Wu CJ. | Acta Agrinomica Sinica. | 2001. 27: 974-979. |
| 66 | C | [J] | Zou CM, Wang GX, HuXD, Zhang YL, Xue LL, Anjum SA, Wang LC. | Chinese Journal of Eco-Agriculture. | 2010. 18: 496-500. |
| 67 | E | [J] | Allmaras RR, Nelson WW, Voorhees WB. | Soil Science Society of America Journal. | 1975. 39: 771-777. |
| 68 | E | [J] | Allmaras RR, Nelson WW. | Soil Science Society of America Journal. | 1971. 35: 974-980. |
| 69 | E | [J] | Anderson EL. | Plant and Soil. | 1986. 95: 293-296. |
| 70 | E | [J] | Anderson EL. | Plant and Soil. | 1988. 108: 245-251. |
| 71 | E | [J] | [Basamba](http://www.tandfonline.com/action/doSearch?action=runSearch&type=advanced&result=true&prevSearch=%2Bauthorsfield%3A(Basamba%2C+T.A.)) TA, [Amézquita](http://www.tandfonline.com/action/doSearch?action=runSearch&type=advanced&result=true&prevSearch=%2Bauthorsfield%3A(Amézquita%2C+E.)) E, [Singh](http://www.tandfonline.com/action/doSearch?action=runSearch&type=advanced&result=true&prevSearch=%2Bauthorsfield%3A(Singh%2C+B.R.)) BR, [Rao](http://www.tandfonline.com/action/doSearch?action=runSearch&type=advanced&result=true&prevSearch=%2Bauthorsfield%3A(Rao%2C+I.M.)) IM. | Acta Agriculturae Scandinavica Section B-Soil and Plant Science. | 2006. 56: 255-262. |
| 72 | E | [J] | Bayuelo-Jiménez JS, Gallardo-Valdéz M, Pérez-Decelis VA, Magdaleno-Armas L, Ochoa I, Lynch JP. | Field Crops Research. | 2011. 121: 350-362. |
| 73 | E | [J] | Bittman S, Liu A, Hunt DE, Forge TA, Kowalenko CG, Chantigny MH, Buckley K. | Journal of Environmental Quality. | 2012. 41: 582-591. |
| 74 | E | [J] | Bray JR, Lawrence DB, Pearson LC. | Oikos. | 1959. 10: 38-49. |
| 75 | E | [J] | Buyanovsky GA, Wagner GH. | Plant and Soil. | 1986. 93: 57-65. |
| 76 | E | [J] | Crozier [CR](http://www.tandfonline.com/action/doSearch?action=runSearch&type=advanced&result=true&prevSearch=%2Bauthorsfield%3A(Crozier%2C+Carl+R.)), King [LD](http://www.tandfonline.com/action/doSearch?action=runSearch&type=advanced&result=true&prevSearch=%2Bauthorsfield%3A(King%2C+Larry+D.)). | Communications in Soil Science and Plant Analysis. | 1993. 24: 1127-1138. |
| 77 | E | [C] | Devereux AF, Fukai S, Hulugalle NR. | Proceedings of 14th Agronomy Conference 20018, 21-25 September 2008, Adelaide, South Australia. | 2008. |
| 78 | E | [J] | Durieux RP, Kamprath EJ, Jackson WA, Moll RH. | Agronomy Journal. | 1994. 86: 958-962. |
| 79 | E | [J] | Eghball B, Maranville JW. | Agronomy Journal. | 1993. 85: 147-152. |
| 80 | E | [J] | Endale DM, Schomberga HH, Fishera DS, Jenkinsa MB, Sharpea RR, Cabrerab ML. | Agronomy Journal. | 2008. 100: 1401-1408. |
| 81 | E | [J] | Follett RF, Allmaras RR, Reichman GA. | Agronomy Journal. | 1974. 66: 288-292. |
| 82 | E | [J] | Foth HD. | Agronomy Journal. | 1962. 54: 49-52. |
| 83 | E | [J] | Godfrey LD, Meinke LJ, Wright RJ. | Journal of Economic Entomology. | 1993. 86: 1557-1573. |
| 84 | E | [J] | Hébert Y, Guingo E, Loudet O. | Crop Science. | 2001. 41: 363-371. |
| 85 | E | [J] | [Ibrikci](http://www.tandfonline.com/action/doSearch?action=runSearch&type=advanced&result=true&prevSearch=%2Bauthorsfield%3A(Ibrikci%2C+H.)) H, [Ulger](http://www.tandfonline.com/action/doSearch?action=runSearch&type=advanced&result=true&prevSearch=%2Bauthorsfield%3A(Ulger%2C+A.+C.)) AC, [Cakir](http://www.tandfonline.com/action/doSearch?action=runSearch&type=advanced&result=true&prevSearch=%2Bauthorsfield%3A(Cakir%2C+B.)) B, [Buyuk](http://www.tandfonline.com/action/doSearch?action=runSearch&type=advanced&result=true&prevSearch=%2Bauthorsfield%3A(Buyuk%2C+G.)) G, [Guzel](http://www.tandfonline.com/action/doSearch?action=runSearch&type=advanced&result=true&prevSearch=%2Bauthorsfield%3A(Guzel%2C+N.)) N. | Journal of Plant Nutrition. | 1998. 21: 1943-1954. |
| 86 | E | [J] | Karunatilake U, van Es HM, Schindelbeck RR. | Soil and Tillage Research. | 2000. 55: 31-42. |
| 87 | E | [J] | Kaspar TC, Brown HJ, Kassmeyer EM. | Soil Science Society of America Journal. | 1991. 55: 1390-1394. |
| 88 | E | [J] | Kaspar TC, Crosbie TM, Cruse RM, Erbach DC, Timmons DR, Potter KN. | Agronomy Journal. | 1987. 79: 477-481. |
| 89 | E | [J] | Kindu M, Roland JB, Bashir J. | Plant and Soil. | 1997. 188: 319-327. |
| 90 | E | [J] | Kuchenbuch RO, Gerke HH, Buczko U. | Plant and Soil. | 2009. 315: 297-314. |
| 91 | E | [J] | Lizaso JI, Melendez LM, Ramirez R. | Journal of Plant Nutrition. | 2001. 24: 979-995. |
| 92 | E | [C] | Long OH. | University of Tennessee Agricultural Experiment Station Bull. | 1959. p 41. |
| 93 | E | [J] | Ma BL, Meloche F, Wei L. | Field Crops Research. | 2009. 111: 189-196. |
| 94 | E | [J] | Maizlish NA, Fritton DD, Kendal PWA. | Agronomy Journal. | 1980. 72: 25-30. |
| 95 | E | [J] | Mengel DB, Barber SA. | Agronomy Journal. | 1974. 66: 341-344. |
| 96 | E | [J] | Nelson WW, Allmaras RR. | Agronomy Journal. | 1969. 61: 751-754. |
| 97 | E | [J] | Ovington JD, Heitkamp S, Lawrence DB. | Ecology. | 1963. 44, 52-63. |
| 98 | E | [J] | Pan B, Bai YM, Leibovitch S, Smith DL. | European Journal of Agronomy. | 1999. 11: 179-186. |
| 99 | E | [J] | Piper [EL](http://www.sciencedirect.com/science?_ob=RedirectURL&_method=outwardLink&_partnerName=27983&_origin=article&_zone=art_page&_linkType=scopusAuthorDocuments&_targetURL=http%3A%2F%2Fwww.scopus.com%2Fscopus%2Finward%2Fauthor.url%3FpartnerID%3D10%26rel%3D3.0.0%26sortField%3Dcited%26sortOrder%3Dasc%26author%3DPiper,%2520Ernest%2520L.%26authorID%3D7003283342%26md5%3Dc71de76ba7d3ba45d6c6280797c71719&_acct=C000053031&_version=1&_userid=1479053&md5=5fc8a32e1d8b0c79a97946918776e3a8), Weiss [A.](http://www.sciencedirect.com/science?_ob=RedirectURL&_method=outwardLink&_partnerName=27983&_origin=article&_zone=art_page&_linkType=scopusAuthorDocuments&_targetURL=http%3A%2F%2Fwww.scopus.com%2Fscopus%2Finward%2Fauthor.url%3FpartnerID%3D10%26rel%3D3.0.0%26sortField%3Dcited%26sortOrder%3Dasc%26author%3DWeiss,%2520Albert%26authorID%3D7402247757%26md5%3Df718cc82a64a18e7088bcdab7cc26831&_acct=C000053031&_version=1&_userid=1479053&md5=26b7658cb164c2c12499a8c71084ce0c) | [Field Crops Research](http://www.sciencedirect.com/science/journal/03784290). | 1993. 31: 145-153. |
| 100 | E | [J] | Rogers HH, Bingham GE, Cure JD, Smith JM, Surano KA. | Journal of Environmental Quality. | 1983. 12: 569-574. |
| 101 | E | [J] | Roth GW, Calvin DD, Lueloff SM. | Agronomy Journal. | 1995. 87: 189-193. |
| 102 | E | [J] | Shamoot S, McDonald L, Bartholomew WV. | Soil Science Society of America Journal. | 1968. 32: 817-820. |
| 103 | E | [J] | Sidiras N, Kendristakis E. | Journal of Agronomy and Crop Science. | 1997. 178: 141-147. |
| 104 | E | [J] | Sierra J, Noël C, Dufour L, Ozier-Lafontaine H, Welcker C, Desfontaines L. | Plant and Soil. | 2003. 252: 215-226. |
| 105 | E | [J] | Thom ER, Watkin BR. | New Zealand Journal of Experimental Agriculture. | 1978. 6: 29-38. |
| 106 | E | [J] | Vamerali T, Saccomani M, Bona S, Mosca G, Guarise M, Ganis A. | Plant and Soil. | 2003. 255: 157-167. |

C and E in the language column denoted Chinese and English, respectively;

J and C in the type column denoted journals and conferences, respectively.

**Table B. Monthly rainfall during the maize growing season in 2011 and 2012, and additional rainfall data from 2007 to 2010 in the experimental station.**

| Year | May | June | July | August | September | Total rainfall (mm) |
| --- | --- | --- | --- | --- | --- | --- |
| 2011 | 13 | 119 | 466 | 63 | 0 | 661 |
| 2012 | 35 | 118 | 310 | 22 | 118 | 603 |
| 2007 | 54 | 75 | 183 | 59 | 57 | 428 |
| 2008 | 56 | 112 | 157 | 213 | 71 | 608 |
| 2009 | 11 | 39 | 75 | 27 | 65 | 216 |
| 2010 | 23 | 123 | 52 | 174 | 67 | 439 |
